# The neurotropic schistosome *vs* experimental autoimmune encephalomyelitis: are there any winners?

**DOI:** 10.1017/S0031182024000210

**Published:** 2024-04

**Authors:** Barbora Šmídová, Martin Majer, Jan Novák, Alena Revalová, Petr Horák, Tomáš Macháček

**Affiliations:** 1 Department of Parasitology, Faculty of Science, Charles University, Prague, Czechia; 2 Institute of Immunology and Microbiology, First Faculty of Medicine, Charles University and General University Hospital in Prague, Prague, Czechia

**Keywords:** demyelination, EAE, eosinophilia, experimental autoimmune encephalomyelitis, IFN-*γ*, neurotropic parasite, neurotropic schistosome, *Trichobilharzia regenti*

## Abstract

The incidences of multiple sclerosis have risen worldwide, yet neither the trigger nor efficient treatment is known. Some research is dedicated to looking for treatment by parasites, mainly by helminths. However, little is known about the effect of helminths that infect the nervous system. Therefore, we chose the neurotropic avian schistosome *Trichobilharzia regenti*, which strongly promotes M2 polarization and tissue repair in the central nervous system, and we tested its effect on the course of experimental autoimmune encephalomyelitis (EAE) in mice. Surprisingly, the symptoms of EAE tended to worsen after the infection with *T. regenti.* The infection did not stimulate tissue repair, as indicated by the similar level of demyelination. Eosinophils heavily infiltrated the infected tissue, and the microglia number increased as well. Furthermore, splenocytes from *T. regenti*-infected EAE mice produced more interferon (IFN)-*γ* than splenocytes from EAE mice after stimulation with myelin oligodendrocyte glycoprotein. Our research indicates that the combination of increased eosinophil numbers and production of IFN-*γ* tends to worsen the EAE symptoms. Moreover, the data highlight the importance of considering the direct effect of the parasite on the tissue, as the migrating parasite may further tissue damage and make tissue repair even more difficult.

## Graphical abstract

**Figure:**
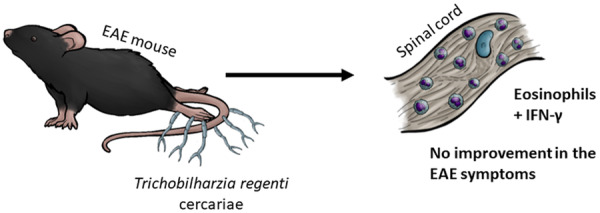

## Introduction

Multiple sclerosis (MS) is a lifestyle autoimmune disease of the central nervous system (CNS) with rising incidence and usual onset at the age of 30–40 years (Walton *et al*., 2020). Neuroinflammation and neuronal damage are driven chiefly by autoreactive antibodies and cytotoxic CD8^+^ T cells in human MS or by T helper cells (Th1 and Th17) in experimental autoimmune encephalomyelitis (EAE), the mouse model of the disease (Dendrou *et al*., 2015; Attfield *et al*., 2022). As the cause of MS remains to be definitely determined, the treatment is primarily symptomatic. Considering the autoimmune nature of MS, targeting the immunopathological processes seems to be a prospective way of mitigating the disease outcome (Tintore *et al*., 2019; Hauser and Cree, 2020; Wiendl *et al*., 2021). While searching for novel MS treatments, the importance of alternatively activated (M2) macrophages has been discovered as they may counteract neuroinflammation and promote tissue recovery (Miron *et al*., 2013). For example, lenalidomide, one of the candidate MS drugs, directly promotes M2 polarization, which leads to reduced CNS infiltration by Th1 and Th17 and decreased tissue demyelination (Weng *et al*., 2018). Other M2-promoting drugs also proved efficient against EAE (Che *et al*., 2022). The beneficial effects of M2 polarization were also confirmed by the adoptive transfer of M2 macrophages (Chu *et al*., 2021), indisputably identifying the M2 pathway as a promising treatment candidate.

To enable survival in the host and reduce tissue pathology, parasitic helminths induce anti-inflammatory (Th2/M2) or regulatory (Treg) host immune milieu (Maizels *et al*., 2018). Hence, their interaction and possible protective effects against MS have been explored in the mouse EAE model. For example, infection of mice with *Heligmosomoides polygyrus*, *Taenia crassiceps* or *Schistosoma mansoni* mitigated the course and severity of EAE by promoting the Th2 response counteracting the neuroinflammatory processes (Dixit *et al*., 2017; Charabati *et al*., 2020). Moreover, Finlay *et al*. (2016) demonstrated the protective effect mediated by interleukin (IL)-5 and eosinophils in EAE mice treated with excretory–secretory products of *Fasciola hepatica*. A similar observation, supporting the role of IL-5 in alleviating EAE symptoms and inducing CNS-protective Treg cells, comes from mice infected with *Nippostrongylus brasiliensis* (Tran *et al*., 2017). Importantly, although not strong, certain positive therapeutic effects were also reported from human MS patients infected with hookworms (Tanasescu *et al*., 2020). These observations indicate that helminths may initiate various mechanisms that could improve the course of MS/EAE.

To explore the diversity of helminth–host interactions under the EAE conditions, we selected the neurotropic avian schistosome *Trichobilharzia regenti*. Contrary to human schistosomes, it migrates through the CNS, being well-adapted to survive in the nervous tissue (Leontovyč *et al*., 2016). In the incidental murine host, it causes mild, non-fatal pathology and is eliminated within 21 days post-infection (Horák *et al*., 1999; Macháček *et al*., 2022). In the spinal cord of experimentally infected mice, the migrating parasites induce an influx of eosinophils and trigger a short-term M1 polarization (Macháček *et al*., 2020). The latter is, however, overwhelmed by massive upregulation of M2 markers no later than 7 days post-infection (Macháček *et al*., 2022). Indeed, M2-canonical IL-4 and arginase are produced in the vicinity of the parasites, with arginase being the most abundant protein in the parasite-surrounding host tissue. For such M2-promoting properties, lasting at least up to 21 days post-infection (Macháček *et al*., 2022), we chose *T. regenti* as a suitable candidate for testing its effects on the course of EAE in mice.

## Materials and methods

### Animal housing

Female C57BL/6JOlaHsd (ENVIGO) mice were housed in the Centre for Experimental Biomodels (Charles University, First Faculty of Medicine) under specific pathogen-free conditions according to the recommendations of the Federation of European Laboratory Animal Science Associations. Mice had unlimited access to food and water.

### Experimental design

**Figure 1.**
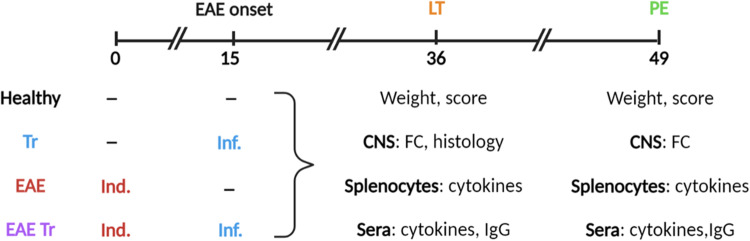
Experimental design. LT, long-term effect; PE, persisting effect; Tr, *Trichobilharzia regenti*; Inf., infection; Ind., induction; FC, flow cytometry.

Eight-week-old mice were randomly divided into 4 groups and subjected to EAE induction and/or *T. regenti* (Tr) infection (Fig. 1). The number of mice per group was *n* = 7 for groups ‘EAE Tr’ and ‘EAE’; *n* = 3 for groups ‘Tr’ and ‘healthy’. The mice were weighed during the whole experiment, and the clinical score was recorded according to Hooke Laboratories (n.d.) (short version is given in Table 1). If a mouse scored ‘4’ for 2 consecutive days, it was euthanized for ethical reasons and scored ‘5’ for the rest of the experiment. This was necessary to perform with 2 ‘EAE’ mice and 1 ‘EAE Tr’ mouse in the long-term (LT) experiment with 1000 cercariae and with 2 ‘EAE Tr’ mice and 1 EAE mouse in the persisting effect (PE) experiment. The samples were harvested on days 36, when the residuals of the parasites are still present in the spinal cord, or 49, when the spinal cord is completely cleared, to examine the LT infection or PE possibly lingering after parasite clearance, respectively.

**Table 1.** Scoring table of clinical symptoms of EAE

Score	Clinical observations
0.0	No obvious changes in motor function compared to non-immunized mice.
0.5	Tip of the tail is limp.
1.0	Limp tail.
1.5	Limp tail and hind leg inhibition.
2.0	Limp tail and weakness of hind legs.
2.5	Limp tail and dragging of hind legs.
3.0	Limp tail and complete paralysis of hind legs.
3.5	Limp tail and complete paralysis of hind legs. Mouse is moving around the cage, but when placed on its side, is unable to right itself.
4.0	Limp tail, complete hind leg and partial front leg paralysis.
4.5	Complete hind and partial front leg paralysis, no movement around the cage.
5.0	Death.

Adapted from Hooke Laboratories (n.d.).

### EAE induction

EAE was induced in ‘EAE’ and ‘EAE Tr’ mice according to Novák *et al*. (2022) on day 0. Briefly, myelin oligodendrocyte glycoprotein-derived 35–55 amino acid peptide (MOG; 400 μg per mouse; APIGENEX, Prague, Czechia) was mixed with complete Freund's adjuvant containing 1 mg mL^−1^ *Mycobacterium tuberculosis* (Sigma Aldrich, St. Louis, USA) in a 1:1 ratio and injected s.c. in 2 places just above the hip joints near the spine. Pertussis toxin (500 ng per mouse; Institute of Microbiology, Czech Academy of Sciences) was injected i.p. on days 0 and 2 of the experiment.

### *Trichobilharzia regenti* infection

With the first symptoms of EAE on day 15, ‘Tr’ and ‘EAE Tr’ mice were infected with *T. regenti* according to a protocol routinely used in our laboratory (Macháček *et al*., 2022). Briefly, cercariae shedding from *Radix* sp. snails were collected and counted, and mice were exposed to either 400 or 1000 cercariae in 50 mL of water in the dark for 1 h.

### Flow cytometry of the CNS

Mice were anaesthetized with Isoflurin (Vetpharma Animal Health, Barcelona, Spain) and transcardially perfused with phosphate-buffered saline (PBS). Cell suspension from the entire CNS was prepared according to Macháček *et al*. (2022). Briefly, the tissue was extracted, gently mechanically homogenized and filtered through a 70 μm cell strainer. Myelin was separated from the cells using 30/70% Percoll (GE Healthcare, Chicago, USA) gradient centrifugation and the cells were collected from the 30/70% interphase. They were washed with PBS, treated with anti-CD16/32 antibody for 10 min, transferred to 96-well U-bottom plates and incubated with a mixture of antibodies against surface markers (CD4, CD11b, F4/80, Ly6G, SiglecF, CD45) at 4°C in the dark for 30 min. The cells were washed with PBS, fixed with a True-Nuclear Transcription Factor Buffer Set (BioLegend, San Diego, USA) and incubated with a mixture of anti-transcription factor antibodies (T-bet, ROR*γ*T, FoxP3) for 30 min in the dark. Redundant antibodies were washed with PBS and the cells were acquired with BC CytoFLEX (Beckman Coulter, Brea, USA). Data were analysed in FlowJo (v10.8.1, BD Biosciences, San Jose, USA). Fluorescence minus one controls were used for CD11b, T-bet and ROR*γ*T. The gating strategy is presented in Fig. S1. Clones and dilutions of the used antibodies are shown in Table S1.

### Histological staining

Mice were anaesthetized with Isoflurin and transcardially perfused with heparinized PBS (10 IU mL^−1^) and ice-cold 4% formaldehyde. The spinal cord was isolated, post-fixed in 4% formaldehyde, dehydrated and embedded in paraffin. Five micrometre slices were prepared and stained with haematoxylin–eosin and Luxol fast blue as follows. First, slides were hydrated with xylene and decreasing alcohol series (100, 96, 75%) and kept for 2 h in 0.1% Luxol fast blue (Solvent Blue 38, Sigma Aldrich) in 96% ethanol with 0.5% glacial acetic acid warmed to 50°C. After cooling, slides were washed with distilled water, differentiated in 0.05% lithium carbonate in distilled water and washed again with distilled water. Then they were stained with Ehrlich's haematoxylin, washed with water, differentiated in acid alcohol (0.2% hydrochloric acid in 70% ethanol) and kept in eosin Y. Finally, slides were washed with water, hydrated in increasing alcohol series (75, 96, 100%) and xylene and mounted with Canada balsam (Sigma Aldrich). Slides were then scanned using Zeiss Axioscan Z1 and analysed in QuPath 0.4.4. Pixel classification was used to determine the dyes, while object classification was used to detect nuclei in the white matter. Both tools were trained specifically for this dataset. Slides used for analysis were then observed and captured using an Olympus BX51 with a camera Olympus DP-2 and processed in GIMP 2.10.36.

### Splenocyte cultivation and cytokine measurement

Mice were anaesthetized with Isoflurin and transcardially perfused with PBS. Spleen was dissected, and splenocyte suspension was prepared according to Majer *et al*. (2020). Briefly, the spleen was homogenized, filtered through a 70 μm cell strainer and got rid of red blood cells with an ACK lysis buffer. Splenocytes were counted using the Countess Automated Cell Counter (ThermoFisher Scientific, Waltham, USA). Two million splenocytes per well (2 million cells mL^−1^) were cultivated in RPMI 1640 supplemented as described in Majer *et al*. (2020) in 12-well plates and stimulated with either MOG (10 or 50 μg mL^−1^), concanavalin A (1.25 μg mL^−1^) or left untreated for 72 h. Concentrations of interferon (IFN)-*γ*, IL-17, IL-1*β*, IL-4, IL-5 and IL-10 were measured in the culture supernatants using ELISA MAX Standard kits (BioLegend) according to the manufacturer's protocol. The concentration of transforming growth factor (TGF)-*β* was measured in the culture media using Mouse TGF-beta 1 DuoSet ELISA (R&D Systems, Minneapolis, USA) according to the manufacturer's protocol.

### Serum analyses

Sera were prepared from blood obtained after anaesthesia with Isoflurin (prior perfusion) and used for cytokine measurement and detection of anti-MOG immunoglobulin G (IgG).

Cytokines IL-4, IL-5, IL-10, IL-17 and IFN-*γ* were measured by Cytokine Bead Array (BD Biosciences) according to the manufacturer's protocol using BD LSR II (BD Biosciences) and analysed in FlowJo (v10.8.1, BD Biosciences).

Anti-MOG IgG levels were detected using an Anti-MOG (35–55) IgG ELISA Kit (Creative Diagnostics, New York City, USA) according to the manufacturer's protocol.

### Statistical analysis

According to the number of factors analysed, either 1- or 2-way analysis of variance (ANOVA) followed by Dunnett's or Šidák's test was used; when the data did not have normal distribution, the Kruskal–Wallis test was used. The survival rate was calculated *via* Kaplan–Meier survival analysis. The analyses were performed in GraphPad Prism (versions 9 and 10), and *P* values <0.05 were considered significant. Data are shown as means with standard deviations or individual values with medians. If not stated otherwise, only significance between the EAE and EAE Tr groups are shown in the graphs.

## Results

### Infection with *T. regenti* tended to worsen the symptoms of EAE in mice

**Figure 2.**
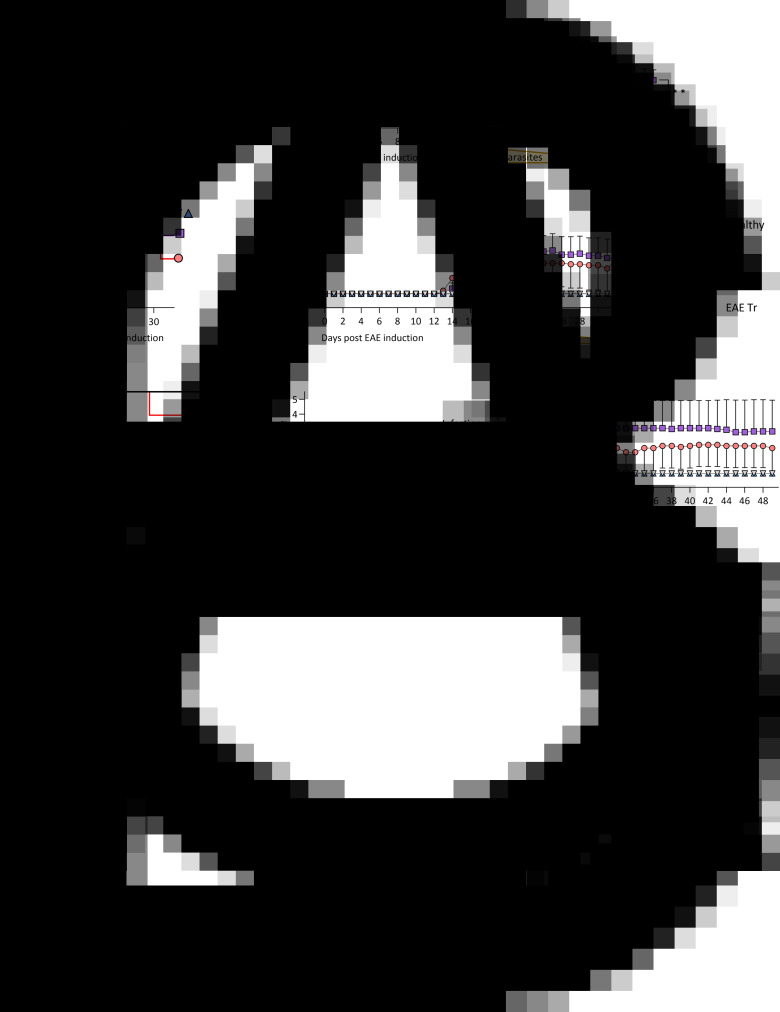
Probability of survival (left column) and clinical score (right column) of experimental mice during long-term infection with either 400 (‘400 LT’, A) or 1000 *T. regenti* cercariae (‘1000 LT’, B) or during persisting effect of the infection with 1000 *T. regenti* cercariae (‘1000 PE’, C). Yellow triangles indicate when and how many migrating parasites can be found in the spinal cord. EAE was induced on day 0 in groups EAE Tr (*n* = 7) and EAE (*n* = 7); EAE Tr and Tr (*n* = 3) mice were infected with *T. regenti* on day 15 (indicated by arrows). Healthy mice (*n* = 3) were used as a control. All mice were scored daily. If a mouse was withdrawn from the experiment for scoring ‘4’ for 2 consecutive days, it was scored 5 for the rest of the experiment. Statistics: Kaplan–Meier survival analysis (left column), mixed effects model of 2-way ANOVA (right column) (**P < 0.01).

Infecting EAE mice with *T. regenti* (EAE Tr) significantly worsened the clinical score of mice in long-term infections with 400 cercariae (400 LT) compared to EAE mice (Fig. 2A). The trend was similar when using 1000 cercariae (1000 LT) (Fig. 2B) and the effect persisted for up to 5 weeks post-infection (Fig. 2C). EAE Tr mice also tended to have a lower probability of survival than EAE mice studied for persisting effect of the infection (1000 PE). On a systemic degree, levels of anti-MOG IgG in sera were slightly elevated only in the EAE group in 1000 PE (Fig. S2). The relative weight of EAE Tr *vs* EAE mice did not change in any experiment (data not shown), and neither did the weight ratio of CNS:body nor spleen:body (Fig. S3).

**Figure 3.**
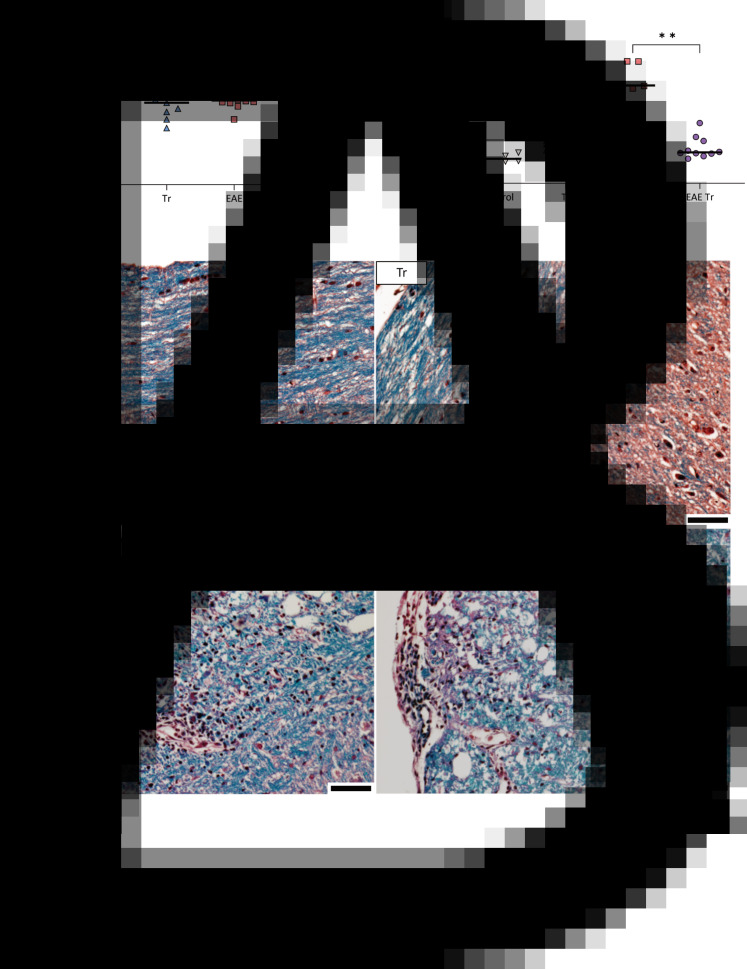
(A) Demyelination and number of nuclei per μm^2^ of white matter in the spinal cord of 1000 LT mice. Pixel and object classification was performed on images of spinal cord sections stained with haematoxylin–eosin and Luxol fast blue. EAE was induced in EAE Tr and EAE mice, groups EAE Tr and Tr were infected with *T. regenti*. 1000 LT, long-term infection with 1000 cercariae. Statistics: 1-way ANOVA (*P < 0.05, **P < 0.01). All significant comparisons are shown for myelin coverage (left graph). (B) Representative photos of the stained sections used for the analysis. Myelin is stained blue. Scale bars: 50 μm. EAE was induced in EAE Tr and EAE mice, groups EAE Tr and Tr were infected with *T. regenti*.

Given that *T. regenti* is found mostly in the white matter up to 21 days post-infection (Macháček *et al*., 2020, 2022), we quantified its myelination and cell infiltration in 1000 LT mice as possible factors contributing to pathology. Representative photos of the spinal cord sections are shown in Fig. 3. A similar level of demyelination was observed in Tr mice as well as in both EAE and EAE Tr mice. However, nuclei density in the white matter was significantly higher in the EAE group compared to the EAE Tr group (Fig. 3), suggesting that the cellular infiltration is stronger in the white matter of the EAE group than in the same tissue of the EAE Tr group.

### *Trichobilharzia regenti* infection increased microglia and eosinophil numbers in the CNS and eosinophil-related IL-5 levels in the serum of EAE Tr mice

**Figure 4.**
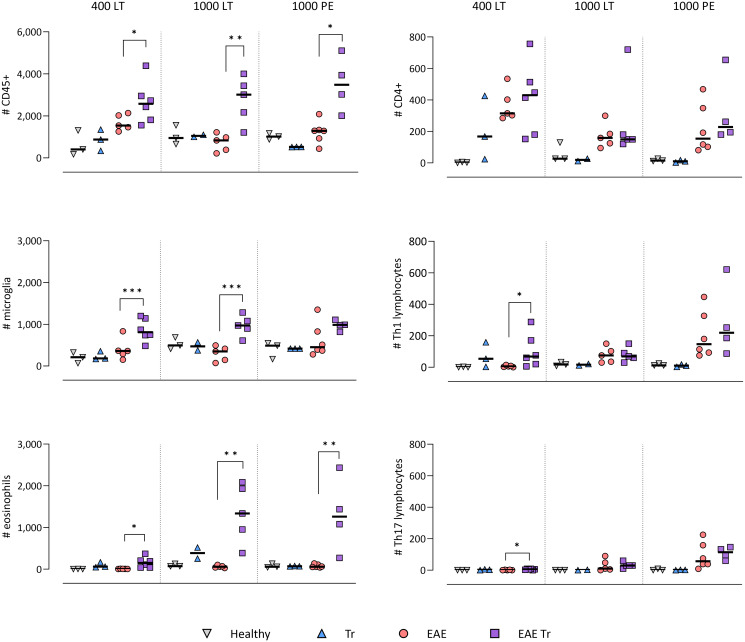
Flow cytometry analysis of overall number of CD45^+^ cells in the CNS (top-left corner), further focused on myeloid cells (left column) and T-lymphocytes (right column). EAE was induced in EAE Tr and EAE mice, groups EAE Tr and Tr were infected with *T. regenti*. Microglia were described as CD45med CD11b^+^; monocytes/macrophages as CD45^+^ CD11b^+^ F4/80^+^; eosinophils as CD45^+^ CD11b^+^ SiglecF^+^; CD4^+^ as CD45^+^ CD4^+^; Th1 lymphocytes as CD45^+^ CD4^+^ Tbet^+^ and Th17 lymphocytes as CD45^+^ CD4^+^ ROR*γ*T^+^. 400 LT, long-term infection with 400 cercariae; 1000 LT, long-term infection with 1000 cercariae; 1000 PE, persisting effect of infection with 1000 cercariae. Statistics: 1-way ANOVA (*P < 0.05, **P < 0.01, ***P < 0.001).

**Figure. 5.**
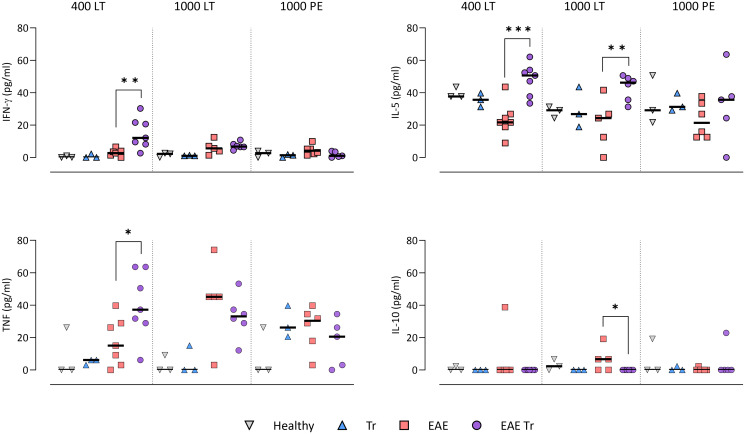
Cytokines measured in sera using cytokine bead assay for flow cytometry. EAE was induced in EAE Tr and EAE mice, groups EAE Tr and Tr were infected with *T. regenti*. 400 LT, long-term infection with 400 cercariae; 1000 LT, long-term infection with 1000 cercariae; 1000 PE, persisting effect of infection with 1000 cercariae. Statistics: 1-way ANOVA (*P < 0.05, **P < 0.01, ***P < 0.001).

**Figure 6.**
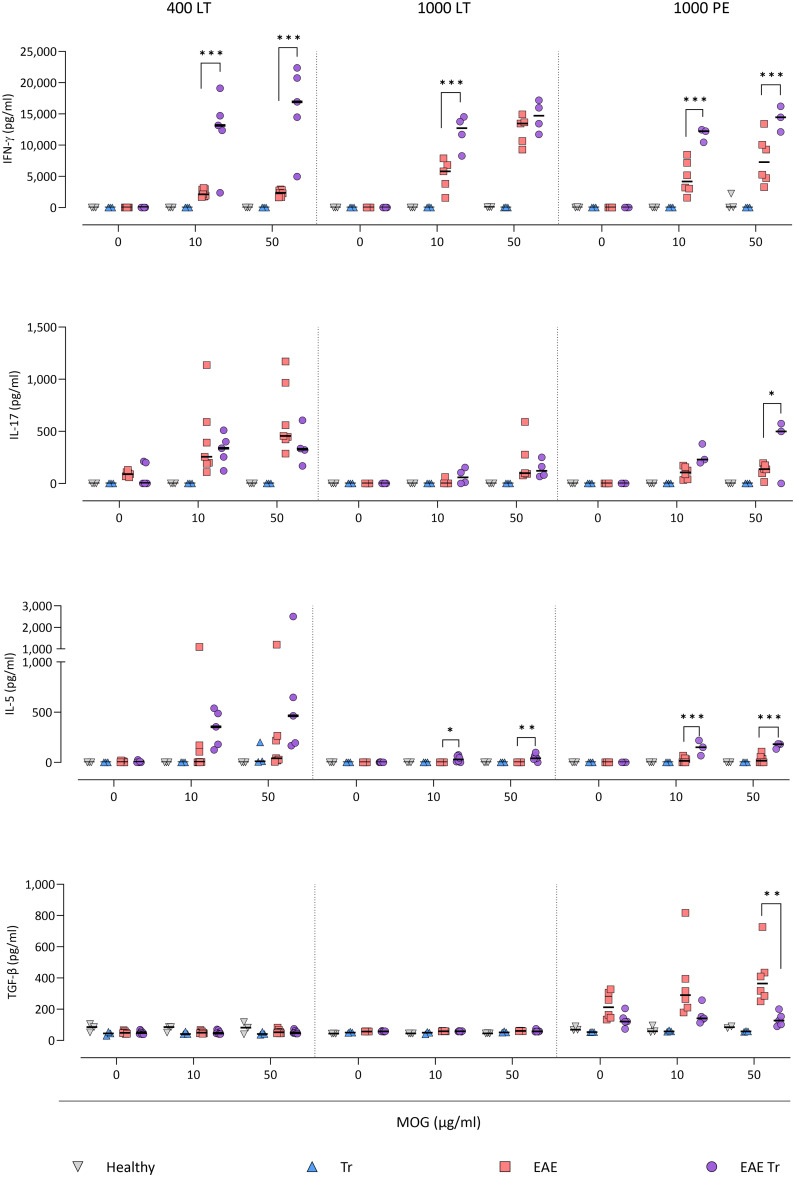
Cytokines measured using ELISA in splenocyte culture media after 72 h stimulation with MOG. EAE was induced in EAE Tr and EAE mice, groups EAE Tr and Tr were infected with *T. regenti*. 400 LT, long-term infection with 400 cercariae; 1000 LT, long-term infection with 1000 cercariae; 1000 PE, persisting effect of infection with 1000 cercariae. Statistics: 1-way ANOVA (*P < 0.05, **P < 0.01, ***P < 0.001).

To further describe the infiltrating immune cells, we performed flow cytometry analysis of the CNS. In timepoints when *T. regenti* is still present in the CNS (Macháček *et al*., 2022), i.e. in 400 LT and 1000 LT experiments, the numbers of total leucocytes and microglia, the resident immune cells, were significantly higher in EAE Tr mice (Fig. 4). However, the relative representation of microglia was decreased in both EAE and EAE Tr mice in all experiments, mainly in favour of T cells or eosinophils (Fig. S4). Eosinophils were the non-resident myeloid cells that most infiltrated the EAE Tr CNS compared to EAE CNS. Their numbers were rather dependent on the initial infection dose than the duration of the infection/EAE, as revealed by the comparison of 1000 LT and 1000 PE groups (Fig. 4). The eosinophil influx in EAE Tr mice was accompanied by an increase in serum IL-5, the cytokine important for eosinophil activation, during long-term infections (Fig. 5). Moreover, splenocytes obtained from EAE Tr mice produced more IL-5 after stimulation with MOG when compared with splenocytes from EAE mice (Fig. 6). Taken together, the data pointed to the importance of eosinophils infiltrating the CNS in EAE Tr mice.

### CD4^+^ cells infiltrating the CNS of EAE Tr mice were mostly Th1 and Th17

Given that T lymphocytes cause the pathology of EAE, we focused on their numbers and phenotype in the CNS, and we also monitored the systemic cytokine profile represented by MOG-stimulated splenocytes. Flow cytometry analysis of the CNS revealed a slightly higher influx of CD4^+^ T cells into the CNS of EAE Tr mice in the 400 LT and 1000 PE groups when compared to EAE mice. Most of them were either Th1 (Tbet^+^) or Th17 (ROR*γ*T^+^). A significant difference in Th1 lymphocytes between EAE Tr and EAE was observed in 400 LT (Fig. 4), and at the same time, the serum levels of Th1-related cytokines, namely IFN-*γ* and TNF, were significantly higher in EAE Tr than in EAE (Fig. 5). Furthermore, EAE Tr splenocytes produced significantly more IFN-*γ* than EAE splenocytes when stimulated with MOG in all experiments (Fig. 6).

As for Th17 cells, they infiltrated the EAE Tr CNS significantly more than the EAE CNS in 400 LT, but the trend was similar in other experiments as well (Fig. 4). Serum levels of IL-17, the prominent Th17-related cytokine, were not significantly different (data not shown). However, in the 1000 PE experiment, EAE Tr splenocytes produced significantly more IL-17 than EAE splenocytes after MOG stimulation (Fig. 6).

Treg cells were also detected *via* flow cytometry of the CNS, but their numbers were similar in all groups in all experiments (data not shown). However, we detected a decrease in serum IL-10 in EAE Tr when compared to EAE in 1000 LT (Fig. 5). Moreover, TGF-*β*, another Treg-related cytokine, was produced less by EAE Tr splenocytes than EAE after MOG stimulation in 1000 PE (Fig. 6). Together the data suggest an important role of IFN-*γ* and propose slight downregulation of Treg response during the infection of EAE mice.

## Discussion

As MS is a disease without an effective definitive cure, the research broadened into studying the possibilities of helminthotherapy. However, most studied helminths do not directly contact the inflamed CNS. Furthermore, in most cases the parasite is introduced before or together with EAE meaning only their prophylactic effect is tested (Dixit *et al.*, 2017). Therefore, we focused on the therapeutic effect of *T. regenti*, the neuropathogenic schistosome capable of inducing a strong M2 response and tissue regenerating process in the CNS (Macháček *et al*., 2022). Although the data overall did not reveal a positive impact of the *T. regenti* infection on the course of the disease, we managed to uncover *T. regenti*-specific relationships between the parasite and the disease.

The general status of EAE mice (mainly the probability of survival) tended to worsen with the infection and the length of the experiment. However, statistical significance was recorded only in EAE mice infected with a lower infection dose, suggesting a dose-dependent effect. Presumably, the low number of parasites reaching the CNS induced only the early-phase inflammation (further described in Macháček *et al*., 2020) but only a few larvae remained in the nervous tissue later in the infection when they induce M2 switch (Macháček *et al*., 2022). On the contrary, a higher infection dose would lead to more larvae and the stimulation of the M2 polarization would be sufficient for counteracting the inflammatory processes. In general, the dose effect is known, e.g. for *F. hepatica* excretory–secretory products (Finlay *et al*., 2016, Lund *et al*., 2016), *T. crassiceps* larvae (Reyes *et al*., 2011) and *Trichuris muris* which triggers Th1 in low-dose infections, but Th2 in high-dose infections (Hayes and Grencis, 2021).

Another explanation of the observed mild worsening of the symptoms might be provided by IFN-*γ*, a cytokine generally associated with inflammation, which was produced in large quantities by splenocytes incubated with MOG peptide. This cytokine supports EAE development (Arellano *et al*., 2015) and can reduce microglial activation under the EAE conditions (Tichauer *et al*., 2023). On the contrary, during the later symptomatic phase of EAE, IFN-*γ* mitigates the disease manifestations (Arellano *et al*., 2015) by supporting TGF-*β* production by microglia (Tichauer *et al*., 2023) and depriving the inflammatory response to further promote Treg function (Furlan *et al*., 2001). Therefore, during the longer-term experiments (such as those presented here), the presence of IFN-*γ* could even be beneficial for the host as it could prevent chronic EAE exacerbation. Further experiments using IFN-*γ*-depleted mice would be necessary to test this hypothesis.

However, the nervous tissue was also infiltrated by many eosinophils which polarize to ‘type 1’ cells when stimulated by IFN-*γ* (Dolitzky *et al*., 2021, 2022). These cells have markers similar to those of M1 macrophages, such as *Cd86*, *Cd53* and *Cd36* (Dolitzky *et al*., 2021). Type 1 eosinophils can produce other inflammatory cytokines (Sakkal *et al*., 2016; Dolitzky *et al*., 2021), and their ability to repair the tissue and stimulate M2 polarization is allayed (Dolitzky *et al*., 2021, 2022). Furthermore, eosinophils can forge even more IFN-*γ* (Lucarini *et al*., 2017) and create a stimulatory loop and inflammatory milieu.

Surprisingly, when the parasite products incite the influx of eosinophils but not the rise of the IFN-*γ* levels, the symptoms of EAE are milder, especially in short-term settings. This effect was observed in EAE mice treated with *F. hepatica* excretory–secretory products (Finlay *et al*., 2016). This brings up 2 questions: (1) Why do eosinophils and IFN-*γ* relieve the EAE symptoms separately but not together, which needs to be further addressed experimentally, and (2) why does *T. regenti*, but not *F. hepatica*, instigate IFN-*γ* production?

To answer the second question, we should consider the tissue damage caused by the parasite migration. For example, the nematode *Toxocara canis*, like *T. regenti*, migrates through the nervous tissue and otherwise has protective immunomodulatory effects (Maizels, 2013), but when combined with EAE, it leads to high levels of IFN-*γ* in the sera and significantly worse symptoms (Novák *et al*., 2022). Indeed, there were also other similarities between these 2 neuropathogens – they both increased the amount of CD4^+^ cells in the CNS, albeit *T. regenti*-triggered increase was not significant. However, in the case of neurotropic unicellular protists (not causing significant tissue damage), such as *Toxoplasma gondii*, the results are opposing. *Toxoplasma gondii* ameliorates the disease symptoms and decreases the levels of inflammatory cytokines (Ham *et al*., 2021), similarly to *F. hepatica* and other non-CNS-resident parasites (Dixit *et al*., 2017). Therefore, we might hypothesize that for experimental EAE helminthotherapy, rather remote immunomodulatory effects associated with non-neurotropic helminths would be beneficial as the neurotropic helminths further damage the nervous tissue and promote inflammation.

To summarize, the data described above lead us to the following hypothesis: when *T. regenti* enters the spinal cord in the symptomatic EAE mice, the early pro-inflammatory response to the parasite (further described in Macháček *et al*., 2020) combines with the ongoing EAE-related neuroinflammation. This is further supported by IFN-*γ* and tissue-infiltrating eosinophils, and the parasite-induced immunomodulatory M2-stimulating properties are not strong enough to overcome this, especially in shorter and low-dose infections. Furthermore, our research highlights the necessity of considering the effect of the migrating parasite itself, especially in a tissue as delicate as the CNS, and supports the strategy of utilizing the immunomodulatory properties of the parasite rather on a systemic level.

## Supporting information

Šmídová et al. supplementary material Šmídová et al. supplementary material

## Data Availability

All data are presented within the article or Supplementary materials.

## Supplementary material

The supplementary material for this article can be found at https://doi.org/10.1017/S0031182024000210
