# Supplementary material for: The neurotropic schistosome vs experimental autoimmune encephalomyelitis: are there any winners?
Source: Parasitology. 2024 Mar 6;151(4):412–20. doi: 10.1017/S0031182024000210 (PMC11044066; doi:10.1017/S0031182024000210)

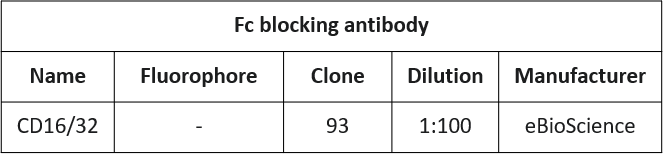

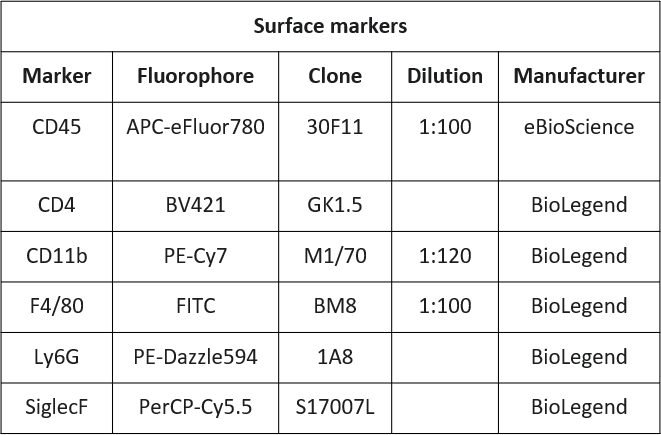

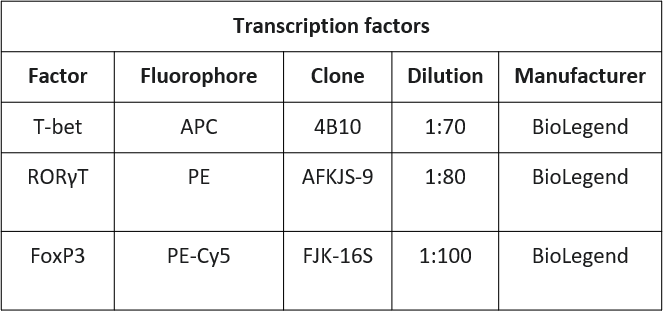
Table S1: Antibodies used for flow cytometry


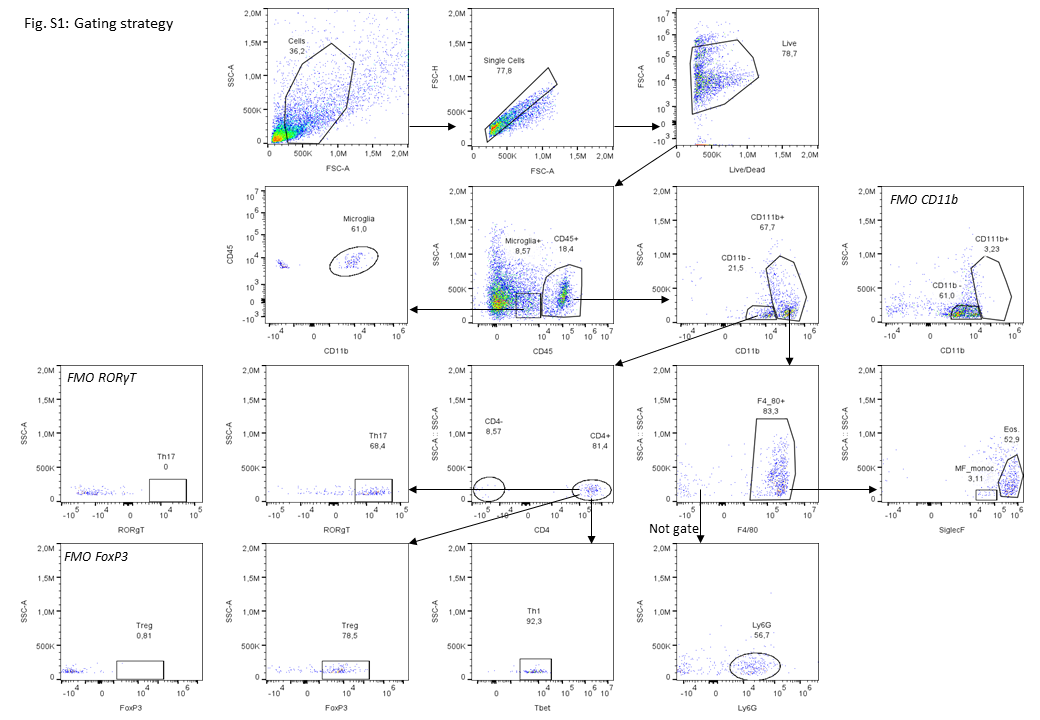


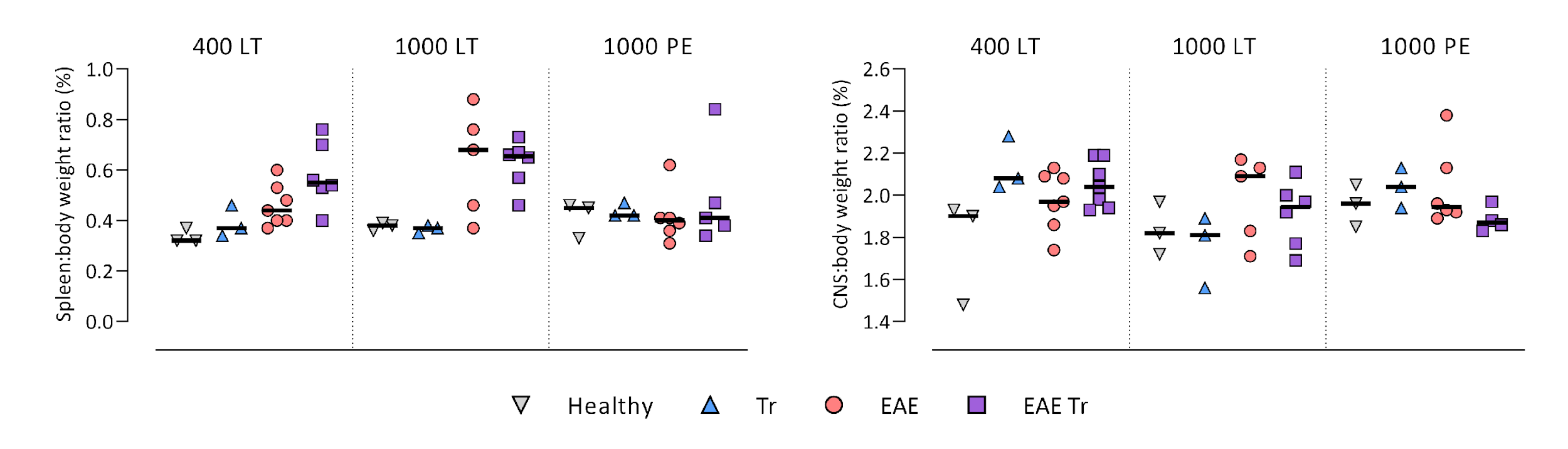


Fig. S3: Weight ratio of spleen to body and CNS to body. EAE was induced to EAE Tr and EAE mice, groups EAE Tr and Tr were infected with *T. regenti*. Abbreviations: 400 LT = long term infection with 400 cercariae; 1000 LT = long term infection with 1000 cercariae; 1000 PE = persisting effect of infection with 1000 cercariae. Statistics: one-way ANOVA.


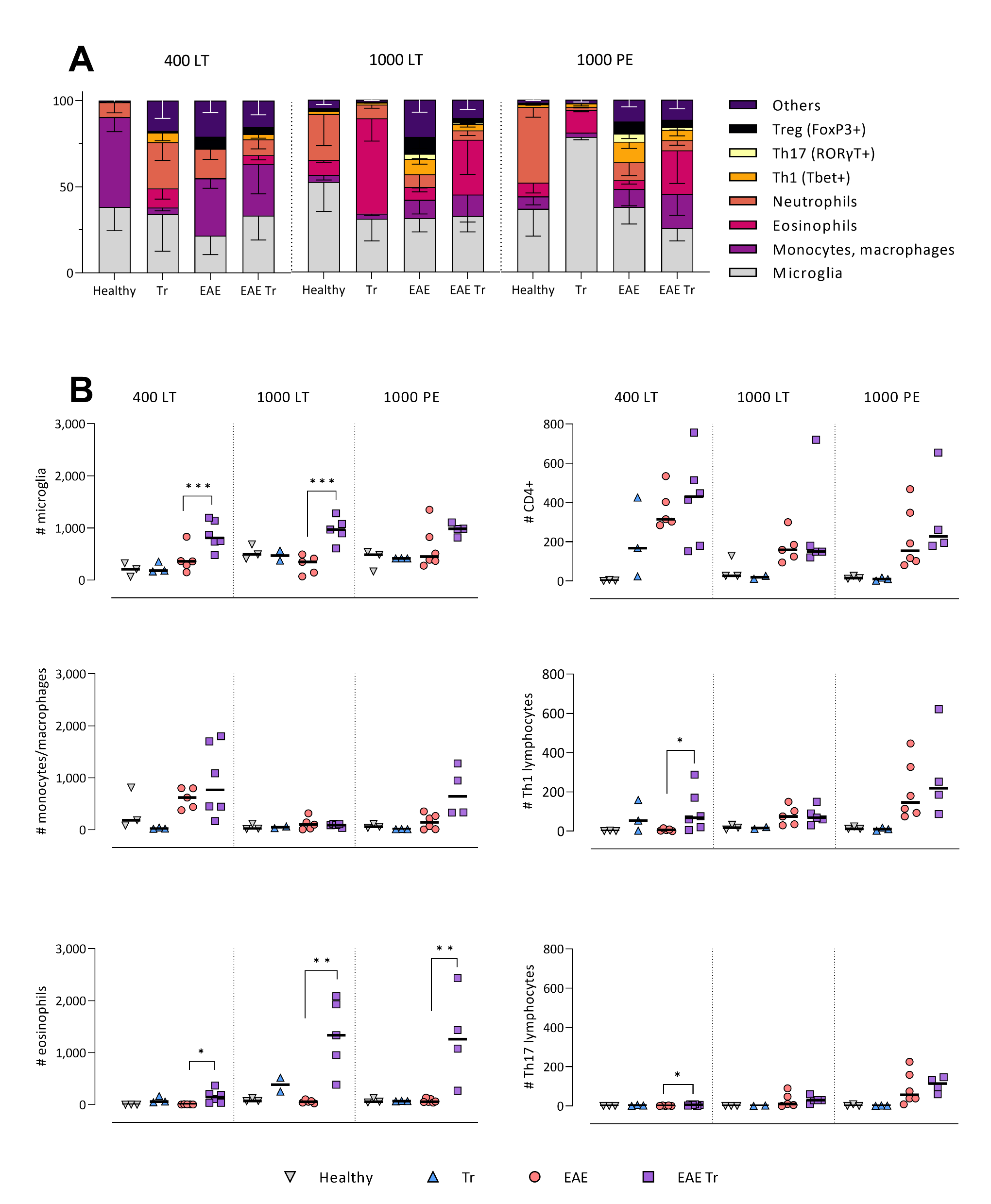


Fig. S4: Relative representation of all examined immune cells in the CNS using flow cytometry. EAE was induced to EAE Tr and EAE mice, groups EAE Tr and Tr were infected with *T. regenti*. Abbreviations: 400 LT = long term infection with 400 cercariae; 1000 LT = long term infection with 1000 cercariae; 1000 PE = persisting effect of infection with 1000 cercariae.

% of CD45^+^

Fig. S2: Levels of anti-MOG IgG in the sera of experimental animals. Abbreviations: 400 LT = long term infection with 400 cercariae; 1000 LT = long term infection with 1000 cercariae; 1000 PE = persisting effect of infection with 1000 cercariae.


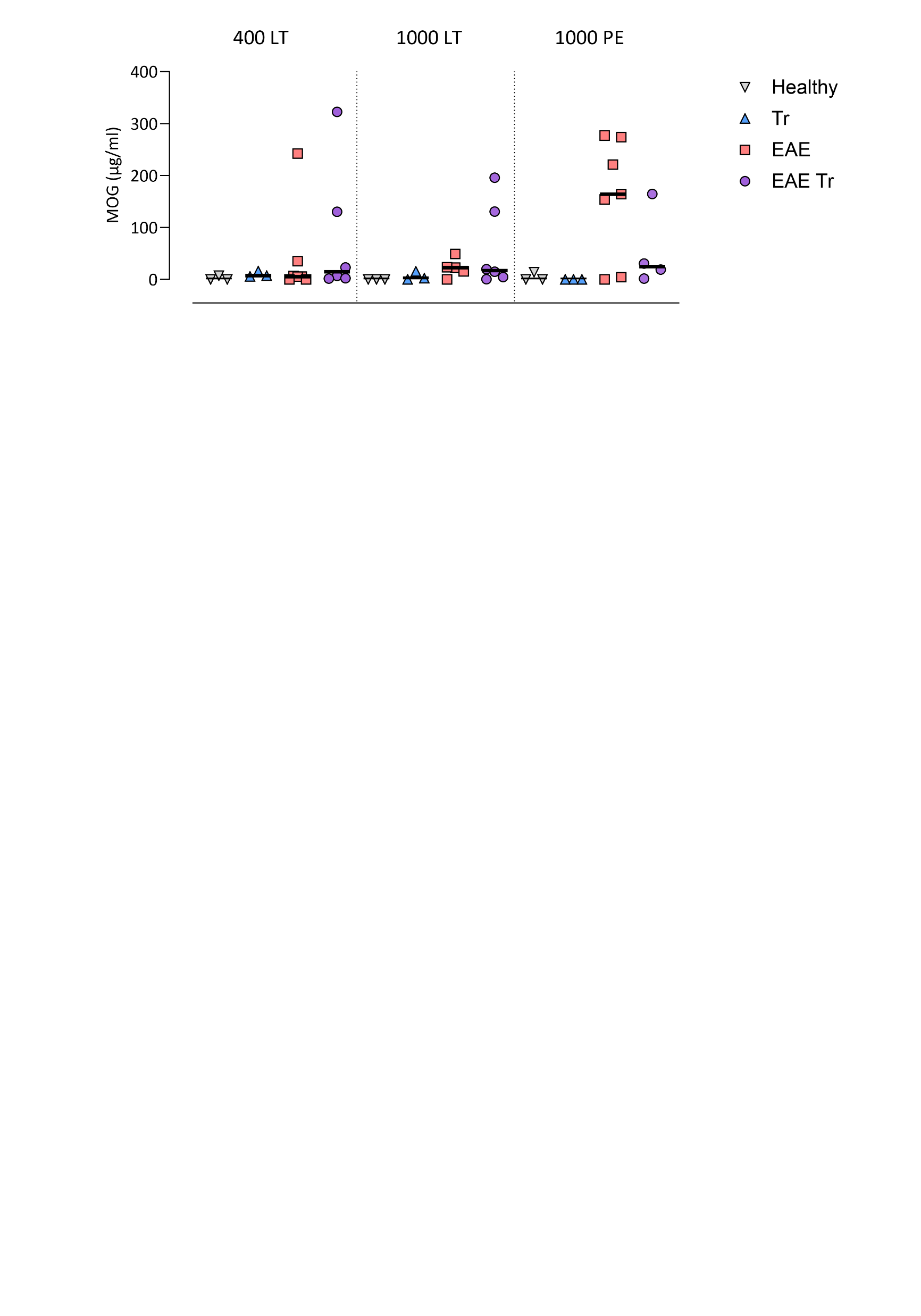

Supplement: Šmídová et al. supplementary material [file S0031182024000210sup001.docx]
